# Healthy lifestyle behaviors among teachers working in public primary schools and affecting factors

**DOI:** 10.3389/fpubh.2024.1382385

**Published:** 2024-04-05

**Authors:** Mukaddes Örs

**Affiliations:** Departmet of Health Management, University of Akdeniz, Antalya, Türkiye

**Keywords:** healthy lifestyle behaviors, health promotion, wellbeing, teacher, primary school

## Abstract

Healthy lifestyle behaviors have been recognized as a key strategy to achieve a policy of health for all. The aim of this study was to determine the levels of health promotion lifestyle behavior among teachers working in public primary schools. The present study also investigated the effects of selected socio-demographic characteristics on these behaviors. The research was designed using the survey model, one of the quantitative research methods. The sample of the research consisted of public primary school teachers (*n* = 372). Research data were collected using the Health-Promotion Lifestyle Profile-II (HPLP-II) scale. As a result of the research, it was put forward that there were statistically significant differences in levels of health promotion lifestyle behaviors based on taking courses on health promotion, and following programs and articles about healthy living (*p* < 0.05). It was revealed that primary teachers' taking courses on health promotion, and following programs and articles affected their healthy lifestyle behaviors. Public primary school teachers' health promotion lifestyle behaviors were found to be moderate. It was found that the primary teachers obtained the highest mean score for the spiritual growth subscale of the health promotion lifestyle behaviors scale; however, the primary school teachers had the lowest mean score for the physical activity subscale, which indicates that they need support in improving their healthy lifestyle. Male teachers had higher mean scores in the physical activity subscale, whereas females had higher mean scores in all other subscales. Training programs to protect and improve the health of teachers should be organized.

## 1 Introduction

Health is defined as a state of complete physical, mental, and social wellbeing and not merely the absence of disease and infirmity (1). Health is one of the most basic human rights; it affects all areas of the life of individuals. Therefore, improving, protecting, and sustaining health is important for all individuals and societies (2, 3). It is stated that human health (the state of complete physical, spiritual, and social wellbeing) depends on lifestyle (up to 70%), heredity (15%), environment (8%−10%), and medicine (8%−10%) (4, 5). Health promotion is a situation that requires people to regulate their lifestyles, keep the behaviors that affect their health under control, and organize their daily living activities by taking responsibility for their own health in order to achieve optimal health conditions (6, 7).

The World Health Organization (WHO) defined its goals and strategies with the slogan “Health for All in the 2000s: 21 Goals in the 21st Century” at the 48th European regional meeting in Copenhagen in 1980. The most obvious aspect of these goals and strategies is the subject of improving health (8, 9). There are three basic strategies for improving health: it is stated as establishing basic conditions for health promotion and advocacy, enabling people to improve their own health, and fostering intersectoral cooperation (3).

Pender (10) defined the health promotion model. The main idea of the model is to create a healthy lifestyle to improve health. Therefore, behaviors need to be changed to be healthy. Pender (10) stated that a healthy lifestyle has two aspects. These are protection and promotion of health. The health promotion model was revised by Pender et al. (11). In the final version of the model, components affecting health-changing behaviors were defined as “individual characteristics and experiences,” “behavior-specific cognitive processes,” and “behavior consequences” (12).

Pender (13) stated that a healthy lifestyle is a component of improving health. A healthy lifestyle is the ability of the individual to control behaviors that may affect health and choose behaviors appropriate to their health status while regulating daily activities. Health behavior is all the behaviors that an individual believes and practices to stay healthy and protect against diseases (14–16). A healthy lifestyle for individuals should not only include protection from diseases but also include behaviors that increase the level of wellbeing throughout life. Healthy lifestyle behaviors contain spiritual development, health responsibility, exercise, nutrition, interpersonal relationships, and stress management (17). It is important for individuals to acquire healthy lifestyle behaviors in order to prevent lifestyle-related diseases and deaths due to these diseases. Acquiring these behaviors is important for preventing chronic diseases, improving the quality of life in the presence of chronic diseases, and promoting healthy aging (18).

It is important to obtain accurate information about health and have appropriate skills in making choices about situations that may affect individuals' health in order to develop healthy lifestyle behaviors (19). Not doing enough physical activity, not having an adequate and balanced diet, and using addictive substances (cigarettes, alcohol, etc.) and health-threatening behaviors are responsible for most of the diseases and deaths associated with chronic diseases (20). According to the global burden of disease study, 34.1 million people died due to preventable risk factors in 2017. One of the five risks that caused death in 2017 was smoking, and the other was high body mass index (BMI) (21). The American Center for Disease Control (CDC) reported that if people demonstrate one or more healthy lifestyle behaviors, their life expectancy will be extended (22).

According to statistics from the World Health Organization, 70%−80% of deaths in developed countries and 40%−50% in underdeveloped countries are caused by diseases that occur due to lifestyle (23, 24). According to the National Burden of Disease and Cost Effectiveness Project data, it is stated that the most important causes of death in Turkey are ischemic heart diseases, cerebrovascular diseases, and chronic obstructive pulmonary disease. A person's own attitude and behavior play a major role in the formation of these non-communicable diseases (25).

Good health boosts successful learning. The wellbeing of each and every student is of utmost importance and is a basic aspect of an efficient education. Lifestyle directly affects the wellbeing of students. Teachers are a significant agent in empowering students with skills for wellbeing and healthy living (26).

According to WHO (27), schools should implement health-promoting policies and practices, such as creating a healthy psychosocial environment for students and staff, equal treatment for all students, policies on drug and alcohol use, tobacco use, first aid, and violence that help prevent or reduce physical, social, and emotional problems.

In Turkey, Article 1 of the Primary Education and Training Law No. 222 defines primary education as an institution that serves the physical, mental, and moral development and upbringing of students. It is possible to achieve this goal by providing a healthy and safe education and training environment (28). Taking necessary precautions for students to acquire knowledge, skills, and habits regarding cleanliness, health, and nutrition is included in the basic laws of primary education (29).

Teachers' behaviors and personality traits affect students' behaviors and personality traits. In this case, teachers with different personalities have different effects on their students. The behaviors of primary school teachers in the classroom affect the students' success in the lesson. An effective and efficient teacher is one who is not only an expert in his or her field at a high level but also one who can adequately explain these competencies to his or her students; in other words, one who provides effective learning and instills behavioral habits. Physical activity-related skills are among the knowledge, skills, and attitudes that primary school teachers should have in subjects such as education, scientific method, anatomy, physiology, psychology, and health. This helps the primary school teacher contribute to the versatile development of students (30).

In school health services, which include all the work to be done to evaluate and improve the health of students and school personnel to ensure a healthy school life and thus to create a healthy society, especially teachers being a good model to students with their own behavior forms the basis for shaping the future lives of students by influencing their behavior (31, 32). However, schools that prepare individuals for life and educate them to develop positive behaviors can also mediate the development of negative behaviors. Unhealthy behaviors acquired in childhood continue in adulthood and put the person's health at risk in the future (33).

Studies most often take into account such lifestyle habits as physical activity, diet, smoking, and hours of sleep (33–36). Many adults often fail to be good role models when they need to exhibit a healthy and balanced lifestyle (37). When it looks at the studies on teachers, in Ak et al. (38) study, 88.8% of teachers smoked at school, in Tokuç and Berberoglu (33) study, the lowest average of health-promoting behaviors was in the exercise dimension, in Gürel et al. (39) study, 80.9% of teachers have inadequate nutrition knowledge. In the study conducted by Kabataş et al. (40) with female teachers, it was determined that 17.6% of the teachers were obese. Studies on healthy lifestyle behaviors in Turkey show that there are important problems. Although many legal regulations and standards have been developed regarding healthy living in schools, it is observed that deficiencies and problems related to healthy living in schools continue in Turkey. In order to develop healthy lifestyle behaviors in primary schools, it is necessary to know the opinions of relevant people and organizations regarding healthy lifestyles.

As a result of the literature review on healthy lifestyle behaviors, it is seen that research in the field of education in Turkey is mostly conducted on health workers, students in schools providing health education, teacher candidates, academicians, and teachers. While studies in this field in Turkey have been applied to different areas, not many studies have been conducted on primary school teachers. For this reason, studies in this field will serve as a resource for future studies.

Teachers have important roles and responsibilities in developing and maintaining healthy lifestyle behaviors among individuals in society. For this reason, teachers need to have knowledge and awareness about health and transform this awareness and knowledge into attitudes and behaviors (41). However, the results of research conducted in Turkey and other countries show that teachers' healthy lifestyle behaviors are not yet at the desired level (33, 37, 42–49). Additionally, when the literature is viewed there are many studies using the “Health-Promotion Lifestyle Profile Scale” in Turkey and other countries. When these studies are evaluated, it is noteworthy that there are studies covering a wide variety of topics, from adolescent mothers (50), to older adult women (51), from chronic disease prevention programs (52), to quality of life in chronic diseases (53, 54), to the evaluation of healthy lifestyle behaviors of students (55, 56), adults (57), and workers (58, 59). However, studies conducted on teachers are quite limited. It is thought that this study will partially eliminate the current deficiency.

It is stated that values related to life and health are acquired at an early age, that primary school teachers have a decisive role in this regard, and that they influence the increase of the general wellbeing level of students and the spread of wellbeing with the education they provide and the positive behaviors they show (60). In light of this information, the problem of this research is to determine the level of health promotion lifestyle behaviors of teachers working in primary schools and also to examine whether socio-demographic characteristics have an effect on their health promotion lifestyle behaviors.

### 1.1 Purpose of the research

The aim of the current study was to determine the levels of healthy lifestyle behavior among teachers working in public primary schools. In addition, it was aimed to determine the effects of some selected variables (gender, marital status, taking courses on health promotion, following programs and articles about healthy living in written and visual media) on healthy lifestyle behaviors. Answers were asked for the following questions:

1. What are the socio-demographic characteristics (gender, marital status, taking courses on health promotion, following programs and articles about healthy living in written and visual media) of the teachers participating in the research?
2. What is the level of health-promotion lifestyle profile (HPLP-II) scale among teachers working in public primary schools?
3. What are the characteristics affecting the health-promotion lifestyle behaviors among teachers?
  (a) Does the level of teachers' health-promotion lifestyle behaviors differ according to their gender?
  (b) Does the level of teachers' health-promotion lifestyle behaviors differ according to their marital status?
  (c) Does the level of teachers' health-promotion lifestyle behaviors differ according to their taking courses on health promotion?
  (d) Does the level of teachers' health-promotion lifestyle behaviors for six components differ according to their following programs and articles about healthy living in written and visual media?

## 2 Materials and methods

### 2.1 The model of the research

The present study was designed using a survey model. The survey model is a research approach that aims to describe a past or present situation or event as it exists (61).

### 2.2 Population and sample

The population of the current study comprised of teachers (*N* = 372) working in diverse branches in 15 public primary schools in Amasya city center in Turkey in the spring term of the 2018–2019 academic year. Amasya is a city, a region in Turkey consisting of around 341.000 inhabitants. All included schools were state-funded, as the vast majority of schools in Turkey are state funded. The research sample consisted of all members of the population. Three hundred fifty teachers were reached due to reasons such as not agreeing to participate in the research, being on leave, sick, and providing incomplete answers to the surveys on the dates the research was conducted. A total of 350 finished questionnaires were returned and analyzed. The convenience sampling technique was used. The data was collected by the researcher, using face-to-face interview techniques in the school environment between June 19 and July 17, 2018.

### 2.3 Data collection tools

A Health-Promotion Lifestyle Profile (HPLP-II) Questionnaire and a personal characteristic form were performed to ascertain the level of health promotion lifestyle behaviors among teachers. The personal information form was a four-item questionnaire. It was purposed at determining the sociodemographic characteristics of participants: gender, marital status, taking courses on health promotion, and following programs and articles about healthy living in written and visual media. The instrument of the present study, titled “Health-Promotion Lifestyle Profile (HPLP-II) Scale,” was originally developed by Walker and Hill-Polerecky (62) and adapted to Turkish by Bahar et al. (63). The questionnaire measures health-promoting behaviors related to a person's healthy lifestyle. It is a questionnaire whose validity and reliability have been proven in applications made on different populations.

This scale consists of 52 items covering six subscales: health responsibility (nine items), physical activity (eight items), nutrition (nine items), spiritual growth (nine items), interpersonal relations (nine items), and stress management (eight items). The questionnaire was presented to participants who answered using a four-point Likert-type scale ranging from 1 “never” to 4 “routinely” (1 = never, 2 = sometimes, 3 = often, or 4 = routinely). The maximum point obtainable from the overall HPLP-II scale is 208. The minimum point obtainable on the overall HPLP-II scale is 52. The lowest and highest points that can be obtained from the subscales are: 9–36 for spiritual growth, 9–36 for health responsibility, 8–32 for physical activity, 9–36 for nutrition, 9–36 for interpersonal relationships, and 8–32 for stress management. As the point obtained from the scale increases, the individual's level of implementation of the specified health behaviors increases. All items on the scale are positive; there are no reverse items. The points in the subgroups can be used independently. The total point of all subgroups of the scale gives the healthy lifestyle behaviors score (63).

It was found that the Cronbach's alpha coefficient was 0.94 for the total scale. The Cronbach Alpha internal consistency coefficients ranging from 0.79 to 0.87 were reported for the subscales (62). The Cronbach Alpha reliability coefficient of the scale by Bahar et al. (63) was found to be 0.92. The Cronbach Alpha coefficient was calculated to test the reliability of the measurements of this research for each sub-scale which was found to be 0.729 for the sub-scale of “health responsibility,” 0.839 for the sub-scale of “physical activity,” 0.707 for the sub-scale of “nutrition,” 0.836 for the sub-scale of “spiritual growth,” 0.821 for the sub-scale of “interpersonal relations,” and 0.707 for the sub-scale of “stress management.” It has been stated that the reliability coefficient on a Likert-type scale should be >0.70 (64).

### 2.4 Analysis of the data

In this research, the SPSS-22.0 package program was performed to analyze the data. Descriptive statistics such as frequency and percentage were used to analyze the data provided from the demographic characteristics of the teachers participating in the research. In addition, descriptive statistics such as mean, standard deviation, frequencies, minimum, and maximum points were calculated for the healthy promotion lifestyle profile II subscales. To examine whether the data were normally distributed, the Kolmogorov-Smirnov test and the Shapiro-Wilks test were performed. The results indicated that it is not normally distributed (*p* < 0.05) (65). To check if the distribution of points was normal, it was calculated at the values of skewness and kurtosis. The kurtosis and skewness coefficients are 0 in a normal distribution (66). In the present study, the skewness test value was 2.86, while the kurtosis test value was 0.19. Since the data did not indicate a normal distribution, the non-parametric tests were performed. The statistical significance level for the *p*-value was set as 0.05.

## 3 Results

The socio-demographic characteristics of the respondents are shown in Table 1. 60.86% of the teachers answering the research are women, and 39.14% are men. Almost all of the teachers (91.71%) were married. The rate of teachers who did not take a course in pre-service training on health promotion is 66.86%, and the rate of those who took a course is 33.14%. When the status of following programs and articles about healthy living in written and visual media is examined, the rate of teachers who are not interested at all is 9.43%, those who are interested when they have the opportunity are 37.14%, those who are interested when necessary are 26.86%, those who are constantly interested are 23.43%, and those who are interested due to their profession are 3.14%.

**Table 1:** Participants' sociodemographic characteristics.

**Variable**	**Category**	***n***	**%**
Gender	Male	137	39.14
	Female	213	60.86
	Total	350	100
Marital status	Married	321	91.71
	Single	29	8.29
	Total	350	100
Taking courses related to health promotion	Yes	116	33.14
	No	234	66.86
	Total	350	100
Status of the following programs and articles about healthy living in written and visual media	Never	33	9.43
	Whenever possible	130	37.14
	When it is necessary	94	26.86
	Continually	82	23.43
	Due to my profession	11	3.14
	Total	350	100

In Table 2, the average total points of the health promotion life profile (HPLP-II) were 129.93 (SD 19.91), indicating that the health promotion life behaviors of the teachers were medium level. The sub-dimension average points of the health promotion lifestyle profile scale are respectively: “spiritual growth” subscale mean score is 26.52 (SD 4.80), “interpersonal relations” subscale mean score is 25.38 (SD 4.60), “nutrition” subscale mean score is 21.73 (SD 3.90), “health responsibility” subscale mean score is 20.80 (SD 4.06), “stress management” subscale mean score was 19.37 (SD 3.65) and “physical activity” subscale mean score was 16.45 (SD 4.93). According to the subscales, “spiritual growth” indicated the highest average point of 26.52 (SD 4.80), whereas “physical activity” indicated the lowest average point of 16.13 (SD 4.76).

**Table 2:** Mean, median, minimum, maximum, and standard deviation of the Health Promotion Lifestyle Profile II (HPLP-II) scale and subscales.

**HPLP-II sub-scales**						
	***n***	**Mean**	**Median**	**Minimum**	**Maximum**	**SD**
Health responsibility	350	20.80	20.00	11.00	34.00	4.06
Physical activity	350	16.13	16.00	8.00	32.00	4.76
Nutrition	350	21.73	21.00	13.00	32.00	3.90
Spiritual growth	350	26.52	27.00	13.00	36.00	4.80
Interpersonal relations	350	25.38	25.00	14.00	36.00	4.60
Stress management	350	19.37	19.00	9.00	30.00	3.65
HPLP-II total scores	350	129.93	128.00	71.00	187.00	19.91

Table 3 shows that there was not significant difference between genders and health promotion lifestyle behavior scores. However, the total mean score of male teachers obtained for health promotion lifestyle was found to be 128.64, which was statistically significantly lower than the mean point of female teachers (130.58), *Z* = −1.89, *p* > 0.05, *r* = 0.1. This represents a small effect size for the gender data (66).

**Table 3:** Mann–Whitney *U*-test results of teachers' healthy lifestyle behaviors II (HPLP-II) scores depending on the their gender variable.

**HPLP-II sub-scales**	**Gender**	***n***	**Mean**	**Median**	**Minimum**	**Maximum**	**SD**	**Mann–Whitney** ***U*****-test**			**Effect size**
								**Mean rank**	***Z***	***p***	***r***
Health responsibility	Male	137	20.40	19.00	11.00	34.00	4.65	158.76	−2.345	0.019	0.12
	Female	213	21.05	21.00	13.00	32.00	3.64	184.60			
	Total	350	20.80	20.00	11.00	34.00	4.07				
Physical activity	Male	137	16.86	16.00	8.00	31.00	5.06	187.02	−1.865	0.062	0.09
	Female	213	15.70	15.00	8.00	32.00	4.51	166.47			
	Total	350	16.16	16.00	8.00	32.00	4.76				
Nutrition	Male	137	21.26	21.00	13.00	32.00	4.16	162.00	−1.862	0.063	0.09
	Female	213	22.00	21.00	14.00	30.00	3.71	182.52			
	Total	350	21.71	21.00	13.00	32,00	3.91				
Spiritual growth	Male	137	26.27	27.00	13.00	36.00	5.19	169.06	−0.81	0.418	0.04
	Female	213	26.61	27.00	15.00	36.00	4.50	177.99			
	Total	350	26.48	27.00	13.00	36.00	4.78				
Interpersonal relations	Male	137	24.63	24.50	15.00	36.00	4.63	156.49	−2.681	**0.007**	0.14
	Female	213	25.80	26.00	14.00	35.00	4.50	186.05			
	Total	350	25.34	25.00	14.00	36.00	4.58				
Stress management	Male	137	19.21	18.00	9.00	30.00	4.24	165.10	−1.402	0.161	0.07
	Female	213	19.42	19.00	11.00	28.00	3.20	180.53			
	Total	350	19.34	19.00	9.00	30.00	3.64				
HPLP-II total scores	Male	137	128.64	125.00	71.00	187.00	23.74	161.78	−1.89	0.059	0.1
	Female	213	130.58	129.50	82.00	170.00	17.04	182.66			
	Total	350	129.82	128.00	71.00	187.00	19.92				

A statistically significant difference was found between genders and health responsibility subscale points (*p* < 0.05). The health responsibility mean point of male teachers was found to be 20.40, which was significantly lower than the mean point of female teachers (*M* = 21.05, *Z* = −2.345, *p* < 0.05, *r* = 0.12). This represents a small effect size for the gender data (66) (Table 3). According to these results, it can be said that gender has an impact on points of the health responsibility subscales of healthy promotion lifestyle behaviors.

When the healthy promotion lifestyle behaviors subscales were investigated, a statistically significant difference was found between genders and teachers' physical activity subscale scores (*p* > 0.05). However, the physical activity average point of female teachers was found to be 15.70, which was significantly lower than the average point of male teachers (*M* = 16.86, *Z* = −1.865, *p* > 0.05, *r* = −0.09). This represents a small effect size for the gender data (66) (Table 3).

A statistically significant difference was found between genders in terms of interpersonal relations subscale points (*p* < 0.05). The interpersonal relations average point of male teachers was found to be 24.63, which was significantly lower than the mean score of female teachers (*M* = 25.80, *Z* = −2.681, *p* < 0.05, *r* = 0.14). This represents a small effect size for the gender data (66) (Table 3). According to these results, it can be said that gender has an impact on scores of the interpersonal relations subscales of health promotion lifestyle behaviors.

There was not statistically significant difference between the genders in terms of their points in the “nutrition” (*M* = 21.71, *Z* = −1.862, *p* > 0.05, *r* = 0.09), “spiritual growth” (*M* = 26.48, *z* = 0.81, *p* > 0.05, *r* = 0.04), and “stress management” subscales (*M* = 19.34, *z* = 1.402, *p* > 0.05, *r* = 0.07; Table 3).

In Table 4, there was not significant difference between the health promotion lifestyle behaviors scale and subscale scores and marital status (*p* > 0.05). In other words, whether the teachers participating in the research were married or single did not make a difference on the healthy lifestyle behavior points. According to these findings, it can be said that marital status has not impact on points of the interpersonal relations subscales.

**Table 4:** Mann–Whitney *U*-test results of teachers' healthy lifestyle behaviors II (HPLP-II) scores depending on their marital status variable.

**HPLP-II sub-scales**	**Marital status**	***n***	**Mean**	**Median**	**Minimum**	**Maximum**	**SD**	**Mann–Whitney** ***U*****-test**			**Effect size**
								**Mean rank**	***Z***	***p***	***r***
Health responsibility	Maried	321	20.81	20.00	11.00	34.00	4.10	175.82	−0.2	0.842	0.01
	Single	29	20.76	20.00	16.00	28.00	3.68	171.91			
	Total	350	20.80	20.00	11.00	34.00	4.06				
Physical activity	Maried	321	15.98	16.00	8.00	32.00	4.65	173.09	−1.488	0.137	0.07
	Single	29	17.76	17.00	10.00	31.00	5.62	202.19			
	Total	350	16.13	16.00	8.00	32.00	4.76				
Nutrition	Maried	321	21.72	21.00	13.00	32.00	3.94	175.01	−0.301	0.763	0.01
	Single	29	21.79	21.00	15.00	27.00	3.45	180.90			
	Total	350	21.73	21.00	13.00	32.00	3.90				
Spiritual growth	Maried	321	26.53	27.00	13.00	36.00	4.67	175.61	−0.068	0.946	0.003
	Single	29	26.38	26.00	15.00	36.00	6.10	174.28			
	Total	350	26.52	27.00	13.00	36.00	4.80				
Interpersonal relations	Maried	321	25.43	25.00	14.00	36.00	4.47	176.31	−0.501	0.616	0.02
	Single	29	24.79	25.00	14.00	34.00	5.85	166.50			
	Total	350	25.38	25.00	14.00	36.00	4.60				
Stress management	Maried	321	19.30	19.00	9.00	30.00	3.55	174.32	−0.727	0.467	0.03
	Single	29	20.07	20.00	13.00	30.00	4.64	188.53			
	Total	350	19.7	19.00	9.00	30.00	3.65				
HPLP-II total scores	Maried	321	129.78	128.00	71.00	187.00	19.48	175.42	−0.05	0.96	0.002
	Single	29	131.55	132.00	98.00	185.00	24.51	176.40			
	Total	350	129.93	128.00	71.00	18.00	19.91				

Table 5 shows that, there was a statistically significant difference between taking courses on topics related to health promotion and the health promotion lifestyle behaviors scale (Mdn = 128.00, *p* < 0.05, *Z* = −4.065, *r* = 0.21). It was determined that the total mean point of teachers who took courses on health promotion (*M* = 136.17) was higher than the mean score of teachers who did not take courses (*M* = 126.83). This represents a small effect size (66). According to these results, it can be said that taking courses on topics related to health promotion has an effect on scores of health promotion lifestyle behaviors.

**Table 5:** Mann–Whitney *U*-test results of teachers' healthy promotion lifestyle behaviors II (HPLP-II) scores depending on their taking courses related to health promotion.

**HPLP-II sub-scale**	**Group**	***n***	**Mean**	**Median**	**Minimum**	**Maximum**	**SD**	**Mann–Whitney** ***U*****-test**			**Effect size**
								**Mean rank**	***Z***	***p***	***r***
Health responsibility	Yes	116	21.56	21.00	13.00	34.00	4.27	193.65	−2.37	0.018	0.12
	No	234	20.43	20.00	11.00	30.00	3.90	166.50			
	Total	350	20.80	20.00	11.00	34.00	4.06				
Physical activity	Yes	116	16.97	16.50	8.00	31.00	4.94	193.58	−2.361	0.018	0.12
	No	234	15.72	15.00	8.00	32.00	4.61	166.54			
	Total	350	16.13	16.00	8.00	32.00	4.76				
Nutrition	Yes	116	22.40	22.00	14.00	32.00	3.64	194.44	−2.473	0.013	0.13
	No	234	21.39	20,50	13.00	32.00	3.99	166.11			
	Total	350	21.73	21.00	13,00	32.00	3.90				
Spiritual growth	Yes	116	28.07	28.00	16.00	36.00	4.26	208.80	−4.345	0.0001	0.23
	No	234	25.75	26.00	13.00	36.00	4.87	158.99			
	Total	350	26.52	27.00	13,00	36.00	4.80				
Interpersonal relations	Yes	116	26.71	27.00	17.00	36.00	4.42	202.42	−3.512	0.0001	0.18
	No	234	24.72	25.00	14.00	35.00	4.55	162.16			
	Total	350	25.38	25.00	14.00	36.00	4.60				
Stress management	Yes	116	20.47	20.00	11.00	30.00	3.96	203.72	−3.688	0.0001	0.19
	No	234	18.82	18.00	9.00	29.00	3.36	161.51			
	Total	350	19.37	19.00	9.00	30.00	3.65				
HPLP-II total scores	Yes	116	136.17	136.00	82.00	187.00	20.63	206.72	−4.065	0.0001	0.21
	No	234	126.83	125.50	71.00	176.00	18.83	160.02			
	Total	350	129.93	128.00	71.00	187.00	19.91				

When the healthy promotion lifestyle profile II sub-scales were analyzed, a statistically significant difference was found between taking courses related to health promotion and the “health responsibility” sub-scale points (*p* < 0.05). The “health responsibility” mean point of teachers who took courses on health promotion during their education (*M* = 21.56) was statistically significantly higher compared to the score of the teachers who did not take courses (*M* = 20.43, *Z* = −2.37, *p* < 0.05, *r* = 0.12). This offers a small effect size (66). According to these results, it can be said that taking courses related to health promotion has an effect on points of the health responsibility subscales of healthy promotion lifestyle behaviors.

There was a significant difference between taking courses related to health promotion and “physical activity” sub-scale points (*p* < 0.05). The “physical activity” mean point of teachers who took courses on health promotion issues (*M* = 16.97) was statistically significantly higher compared to the point of teachers who did not take courses (*M* = 15.72), *z* = −2.361, *p* < 0.05, *r* = 0.12 (Table 5). This offers a small effect size (66).

There was a significant difference between taking courses on health promotion and the “nutrition” subscale point (*p* < 0.05). The “nutrition” average point of teachers who did not take courses on health promotion (*M* = 21.39) was lower than the average point of teachers who took courses (*M* = 22.40), *z* = −2.473, *p* < 0.05, *r* = 0.13 (Table 5). This offers a small effect size (66).

There was a significant difference between spiritual growth subscale score averages and taking courses on health promotion subjects (*p* < 0.05). The “spiritual growth” dimension mean score of teachers who did not take courses on health promotion (*M* = 25.75) was significantly lower than the mean point of teachers who took courses (*M* = 28.07), *z* = −4.345, *p* < 0.05, *r* = 0.23; Table 5). This offers a small effect (66).

There was a significant difference between taking courses on health promotion subjects and the “interpersonal relations” subscale mean score (*p* < 0.05). The mean score of the interpersonal relations dimension of teachers who did not take courses on health promotion (*M* = 24.72) was significantly lower than the scores of teachers who took courses (*M* = 26.71, *z* = −3.512, *p* < 0.05, *r* = 0.18; Table 5). This offers a small effect (66).

There was a significant difference between taking courses on health promotion subjects and stress management subscale points (*p* < 0.05). The “stress management” mean point of teachers who did not take courses on health promotion subjects (*M* = 18.82) was significantly lower compared to the mean point of teachers who took courses (*M* = 20.47), *z* = −3.688, *p* < 0.05, *r* = 0.19; Table 5). This offers a small effect (66). Based on these findings, it can be said that taking courses on health promotion subjects has an effect on all of the subscales of the health promotion lifestyle behavior scores of the teachers.

When Table 6 is examined, there is a significant difference between the status of following healthy lifestyle programs and articles in written and visual media in terms of health promotion lifestyle profile scale points (*H* = 50.153, SD = 19.91, *p* < 0.05, *r* = 0.14). The average score of teachers who were never interested in programs and articles about healthy living in written and visual media 122.09 (SD 23.33) was significantly lower compared to the point of those who were interested when they had the opportunity 124.28 (SD 16.56), when necessary 128.43 (SD 17.78), always interested 141.85 (SD 17.95), and by profession 144.09 (SD 31.24; Table 6). Based on these findings, it can be said that following programs and articles about healthy living in written and visual media has an effect on teachers' health promotion lifestyle profile points.

**Table 6:** Kruskal–Wallis *H*-test results of teachers' Health Promotion Lifestyle Profile II (HPLP-II) scale and subscale scores depending on their following programs and articles about healthy living in written and visual media.

**HPLP-II sub-scale**	**Variable**	***n***	**Mean**	**Median**	**Minimum**	**Maximum**	**SD**	**Kruskall–Wallis** ***H*****-test**			**Effect Size**
								**Mean rank**	***H***	***p***	***r***
Health responsibility	1 = never	33	19.33	19.00	11.00	28.00	4.11	137.36	53.118	0.0001	0.15
	2 = possible	130	19.83	19.50	13.00	30.00	3.32	152.17			
	3 = necessary	94	20.02	19.00	11.00	30.00	3.78	157.86			
	4 = constantly	82	23.59	23.00	14.00	34.00	3.91	243.57			
	5 = by profession	11	22.64	26.00	16.00	28.00	5.43	209.05			
	Total	350	20.80	20.00	11.00	34.00	4.06		4-1 4-2 4-3 4-5		
Physical activity	1 = never	33	17.45	16.00	12.00	32.00	5.01	195.05	24.213	0.0001	0.06
	2 = possible	130	14.95	14.50	8.00	26.00	4.00	150.98			
	3 = necessary	94	15.38	15.00	8.00	28.00	4.58	163.89			
	4 = constantly	82	17.80	18.00	8.00	30.00	4.80	213.36			
	5 = by profession	11	20.00	16.00	14.00	31.00	7.18	223.64			
	Total	350	16.13	16.00	8.00	32.00	4.76		2-1 2-4 2-5 3-1 3-4 3-5		
Nutrition	1 = never	33	19.64	19.00	15.00	28.00	3.90	118.24	29.608	0.0001	0.08
	2 = possible	130	21.02	20.50	13.00	32.00	3.50	158.83			
	3 = necessary	94	21.74	21.00	14.00	29.00	3.64	178.11			
	4 = constantly	82	23.43	24.00	14.00	32.00	4.09	216.52			
	5 = by profession	11	23.45	26.00	18.00	28.00	4.18	216.18			
	Total	350	21.73	21.00	13.00	32.00	3.90		1-2 1-3 1-4 1-5 2-4 2-5		
Spiritual growth	1 = never	33	24.58	25.00	15.00	36.00	5.73	136.03	27.704	0.0001	0.07
	2 = possible	130	25.50	26.00	16.00	34.00	4.43	153.11			
	3 = necessary	94	26.53	28.00	13.00	35.00	4.81	178.68			
	4 = constantly	82	28.60	28.50	18.00	36.00	4.00	217.94			
	5 = by profession	11	28.82	28.00	20.00	36.00	5.74	215.00			
	Total	350	26.52	27.00	13.00	36.00	4.80		1-3 1-4 1-5 2-3 2-4 2-5		
Interpersonal relations	1 = never	33	22.73	23.00	14.00	32.00	5.74	129.70	38.36	0.0001	0.10
	2 = possible	130	24.27	24.00	16.00	35.00	4.04	148.91			
	3 = necessary	94	25.59	26.00	15.00	35.00	4.29	179.37			
	4 = constantly	82	27.66	28.00	17.00	36.00	4.06	225.90			
	5 = by profession	11	27.73	28.00	21.00	34.00	5.35	218.36			
	Total	350	25.38	25.00	14.00	36.00	4.60		4-1 4-2 4-3 5-1 5-2 5-3		
Stress management	1 = never	33	18.36	17.00	13.00	29.00	4.05	139.36	23.409	0.0001	0.06
	2 = possible	130	18.70	18.00	13.00	30.00	3.26	156.90			
	3 = necessary	94	19.16	19.00	9.00	29.00	3.46	174.65			
	4 = constantly	82	20.78	21.00	13.00	28.00	3.46	217.34			
	5 = by profession	11	21.45	19.00	15.00	30.00	5.94	199.14			
	Total	350	19.37	19.00	9.00	30.00	3.65		4-1 4-2 4-3 5-1 5-2 5-3		
HPLP-II total scores	1 = never	33	122.09	115.00	94.00	176.00	23.33	130.89	50.153	0.0001	0.14
	2 = possible	130	124.28	123.00	95.00	187.00	16.56	144.44			
	3 = necessary	94	128.43	129.00	71.00	170.00	17.78	176.12			
	4 = constantly	82	141.85	140.00	97.00	176.00	17.95	236.07			
	5 = by profession	11	144.09	146.00	106.00	185.00	31.24	219.50			
	Total	350	129.93	128.00	71.00	187.00	19.91		4-1 4-2 4-3 5-1 5-2 5-3		

A statistically significant difference was revealed between the “physical activity” dimension subscale point average of the teachers participating in the study and their status of following programs and articles about healthy living in written and visual media (*H* = 24.213, SD = 4.76, *p* < 0.05, *r* = 0.06). When the average physical activity subscale score of the teachers answering in the research was compared with their status of following programs and articles about healthy living in written and visual media: never interested 17.45 (SD 5.01), those who were interested whenever possible 14.95 (SD 4.00), those who were interested when necessary 15.38 (SD 4.58, *p* < 0.05), those who were constantly interested 17.80 (SD 4.80), and those who were interested in their profession 20.00 (SD 7.18) subscale points, a significant difference was revealed between the averages (Table 6). Based on these findings, it can be said that following programs and articles about healthy living in written and visual media has an effect on teachers' physical activity subscale scores.

A significant difference was revealed between the “nutrition” dimension subscale point average of the teachers answering in the study and their status of following programs and articles about healthy living in written and visual media (*H* = 29.608, SD = 3.90, *p* < 0.05). The nutrition dimension subscale score average of teachers who never follow programs and articles about healthy living in written and visual media was 19.64 (SD 3.9); those who follow them when they have the opportunity were 21.02 (SD 3.50); those who follow them when necessary were 21.74 (SD 3.64); and those who follow them constantly were 23.43 (SD 4.09); and due to their profession 23.45 (SD 4.18; Table 6). Subscale points showed a statistically significant difference between the averages. Based on these findings, it can be said that following programs and articles about healthy living in written and visual media has an effect on teachers' “nutrition” subscale score.

A significant difference was revealed between the “spiritual growth” subscale mean score of the teachers participating in the research and their following of programs and articles about healthy living in written and visual media (*H* = 27.704, SD = 4.80, *p* < 0.05). The spiritual growth mean score of those who never follow programs and articles about healthy living was 24.58 (SD 5.73), the mean score of those who follow them when they have the opportunity was 25.50 (SD 4.43) and when necessary was 26.53 (SD 4.81), the score of those who follow them constantly was 28.60 (SD 4.00), and due to their profession was 23.45 (SD 4.18) subscale points. A significant difference was revealed between the averages (Table 6). Based on these findings, it can be said that following programs and articles about healthy living in written and visual media has an effect on teachers' “spiritual growth” subscale score.

There was a significant difference between the “interpersonal relations” subscale point of the teachers participating in the research and their following of programs and articles about healthy living in written and visual media (*H* = 38.36, SD = 4.60, *p* < 0.05, *r* = 0.10). The spiritual growth mean score of those who never follow programs and articles about healthy living was 22.73 (SD 5.74), the mean score of those who follow them when they have the opportunity was 24.27 (SD 4.04) and when necessary was 25.59 (SD 4.29), the score of those who follow them constantly was 27.66 (SD 4.06), and due to their profession 27.73 (SD 5.35; Table 6). The subscale points showed a significant difference between the averages. Based on these findings, it can be said that following programs and articles about healthy living in written and visual media has an effect on teachers' “interpersonal relations” subscale score.

There was a significant difference between the stress management subscale points of the teachers participating in the study and their following of programs and articles about healthy living in written and visual media (*H* = 23.409, SD = 3.65, *p* < 0.05, *r* = 0.06). The stress management mean score of those who never follow programs and articles about healthy living was 18.36 (SD 4.05), the mean score of those who follow them when they have the opportunity was 18.70 (SD 3.26) and when necessary was 19.16 (SD 3.46), the score of those who follow them constantly was 20.78 (SD 3.46), and due to their profession 21.45 (SD 5.94; Table 6). The subscale points showed a significant difference between the averages. Based on these findings, it can be said that following programs and articles about healthy living in written and visual media has an effect on teachers' “stress management” subscale score.

## 4 Discussion

The purpose of this research was to determine the levels of health promotion lifestyle behavior among teachers. In addition, the present study examined whether health promotion lifestyle behaviors differ according to selected socio-demographic characteristics such as gender, marital status, taking courses on health promotion, and following programs and articles about healthy living in written and visual media.

In this study, it was found that the overall score of teachers on the health promotion lifestyle behaviors questionnaire was at a medium level. The maximum score that can be obtained from the health promotion lifestyle behaviors questionnaire is 208, and the average health promotion lifestyle behaviors score in the present study was found to be 129.93. In line with this result, it is suggested that the health promotion lifestyle behaviors of the participants should be supported. In some studies conducted in Turkey, the average healthy lifestyle behavior scores were 122.1 ± 19.8 (67), 125.9 ± 17.4 (68), 134.5 ± 17.9 (33), 128.74 ± 18.24 (69), and 144.90 ± 24.07 (70). It has been found that the average score of physical education teachers' healthy lifestyle behavior was 145.7 ± 15.5 (71). Kaya et al. (72) stated that the general score of faculty lecturers on health-promoting lifestyle behaviors was 139.5 ± 18.0, which was found to be higher than this study. In a study conducted by Çebi (73) to determine the healthy lifestyle behaviors of athletes, the total points of the athletes on the healthy lifestyle behavior scale were found to be 135.74 ± 21.46. In studies conducted with the same scale in other countries, health promotion lifestyle behaviors mean scores were found to be lower (58). Rahnavard et al. (74) found that an undesirable lifestyle was detected in 50% of the teachers. Pirzadeh et al. (47) stated that 23% of teachers had a moderately healthy lifestyle. In the study conducted by Seema (26) on teachers working in secondary schools, the average value of healthy lifestyle behaviors was revealed to be 135.9.

Health promotion lifestyle behaviors in the current research were analyzed regarding six dimensions. Regarding the findings of the six dimensions, it has been seen that the highest average point percentage of health promotion lifestyle behaviors was found for teachers responses to spiritual growth component followed by the interpersonal relationships component, while teachers' health promotion lifestyle to physical activity dimension was the lowest. The subscale indicates that teachers need support in physical activity skills. The high spiritual growth score of the teachers participating in the research can be interpreted as their feeling of value and self-appreciation. This situation is extremely important for the development of the profession. Many studies can be found in the literature with similar findings to the current study. It was found that the participants received the highest average point in the spiritual development sub-scale and the lowest mean point in the physical activity sub-scale (2, 37, 75–81). In some other studies, it was found that the highest mean point of the participants was in the interpersonal relations sub-scale, the lowest score was in the physical activity sub-scale (82–85), and in some studies, the lowest sub-scale point was in the coping with stress sub-factor (46, 73). In this study, similar to other studies, it can be said that teachers did not do enough exercise. However, problems arising from changes in individuals' lifestyles, especially sedentary living, are among the most important causes of chronic diseases and deaths today (86). On the other hand, it is thought that the low average scores of teachers on physical activity, as well as coping with stress and health responsibility, may pose a potential risk for various diseases, such as cardiovascular diseases.

The current study found that there was not a statistically significant difference between sex and the total point of health promotion lifestyle behaviors. It was found that male teachers' healthy lifestyle behavior scale scores were significantly lower than those of female teachers. The reason for this difference may be related to the cultural structure and the fact that women look at issues such as health, nutrition, and aesthetics more responsibly than men. This result contradicts with Kafkas et al.'s (71) study in Turkey, which found that there was a significant difference between sex data and overall health promotion lifestyle behaviors. Tabrizi et al.'s (87) comprehensive review revealed that the results of studies on the importance of sex in health-promoting behaviors are not consistent.

The present study found there was not significant difference between the sex and physical activity sub-scale. However, male teachers scored higher on the physical activity scale. This result matched that of Esin (88) and Baltaş (89), who reported that women exercise less. Previous research has reported a significant difference between sex and physical activity. When studies in Turkey and other countries were examined, the physical activity levels of male participants were determined to be higher than those of female participants, consistent with our study, although some of them did not have statistical significance (37, 90, 91). Indeed, the frequency of doing sports or exercising among male teachers was also found to be higher than that of female teachers among secondary school teachers in Austria. Dearden and Sheahan (92) found that women do not want to engage in physical activity, and the reasons for this are individual, family, and social factors such as lack of facilities and equipment, a safe place for walking, and time limitation. The reasons why male teachers' physical activity scores are significantly higher than females are: this may be due to the fact that female teachers have restrictions on going out in the evening because such activities can usually be done in the evening after class schedules; that men have more exercise opportunities in the evening; and that men prefer exercise (especially group sports) to socialize and relieve stress.

The present study found a significant difference between the interpersonal relations sub-scale and the sex variable. The female teachers received higher points on the interpersonal relations sub-scale. The study conducted by Karakoç's (37) found that female teachers received higher scores than male teachers in the interpersonal relations dimension, but these differences were not found to be statistically significant.

In the present study, there was not significant difference between the “health responsibility” sub-scales and sex. Health responsibility means that the individual shows a change in attitude and behavior toward protective behaviors, preventive behaviors, and health-promoting behaviors regarding his own health. It is stated that health responsibility affects the individual's quality of health care (93). An important finding was obtained for teachers who are education workers and should be role models.

In the present study, there was not statistically significant difference between the “physical activity,” “nutrition,” “spiritual growth,” and “stress management” sub-scales with sex. However, the scores of female teachers are higher than those of male teachers. According to the current research's results, it can be said that female teachers who participated in the research were more likely to implement health promotion lifestyle behaviors outside of physical activity.

In this study, there was not significant difference between teachers' health promotion lifestyle behaviors scale, subscales, and their marital status (*p* > 0.05). However, the overall average point of health promotion lifestyle behaviors of married teachers was found to be lower than the average total score of single teachers. Consistent with the results of the present research, some studies in Turkey and other countries have found that there is not statistically difference between healthy lifestyle behaviors and marital status (45, 90). Previous studies have stated a significant difference between marital status and health-promoting lifestyle behaviors, but this was not evidently statistically significant in the present study. This result contradicts the results of the previous research. In the study conducted by Kiliç and Çimen (90), in the sub-scale of health responsibility (*p* = 0.008), the physical activity subscale (*p* =0.037) was revealed to have significant differences in favor of marriage. This can be explained by the fact that marriage imposes more responsibilities on individuals, provides significant social support to spouses, and married people lead a more regular lifestyle.

In the current research, it was found that there was a statistically significant difference between teachers who took courses on topics related to health promotion in terms of the health promotion lifestyle behaviors scale and sub-scales. Healthy lifestyle behaviors scale total point and subscale total point averages were determined to be higher for teachers who took courses on health promotion. This result is congruent with Kostak et al. (46) in Turkey, who reported that the health promotion lifestyle behaviors scores of the primary school teaching students who took courses on health promotion were higher than those of those who did not take courses during their education. In another study conducted with nursing students, the average points of the health responsibility and nutrition sub-scale and the Healthy Lifestyle Behaviors Scale were determined to be significantly higher in those who took courses on health protection and promotion (94).

It is important that teachers taking health-related courses have good healthy lifestyle behaviors, as it shows that the inclusion of health and health promotion courses in the school curriculum is efficient in helping individuals learn healthy lifestyle behaviors and make them a habit. The development of health-related behaviors in general is possible by quitting bad habits or adopting and continuing healthy lifestyle behaviors. It is suggested that in order to provide students with awareness of positive health behaviors and the protection, maintenance, and development of health, health promotion issues should be included more in primary school curricula, and practices aimed at improving health should be implemented in high school and university curricula.

In the current research, the total health promotion lifestyle behaviors score stated a statistically significant difference with teachers' status of following programs and articles about healthy living in written and visual media (*p* < 0.05). It has been found that the total point of teachers who follow healthy life-related programs in written and visual media is higher than the total score of teachers who do not follow them. The present study's result matched that of Üçdal (49), who stated that scores of overall health promotion lifestyle behaviors of physical education teachers showed a significant difference with the status of following programs and articles about healthy living in written and visual media. Üçdal (49) determined that the total score of teachers who follow health-related programs is 146.4000, the total score of teachers who do not follow health programs is 133.0175, and the total score of teachers who sometimes follow health programs is 137.3385. However, in the study conducted by Üçdal (49), no significant difference was determined between the stress management sub-scale and the status of following health-related programs from the media.

According to these findings, the following recommendations have been made for teachers working in primary schools to adopt, implement, and maintain a healthy lifestyle:

In-service training on healthy lifestyles should be organized so that teachers can adopt a healthy lifestyle and apply it to their lives.

Training programs that provide detailed information about the benefits of exercise and encourage exercise should be organized.

Teachers, who have a high risk of constantly encountering stressful situations should be made aware of this situation, how to cope effectively, and how to resolve the events.

It is suggested that different scientific studies be carried out by determining health promotion lifestyle behaviors and working in coordination with the Ministry of Health and the Ministry of National Education.

Considering that male teachers mostly score high in exercise and female teachers score high in nutrition, it is thought that it would be appropriate to create opportunities and possibilities for physical activity for female teachers and to make plans and practices to raise awareness among male teachers about nutrition.

Establishing training programs on health-promoting lifestyle behaviors and making these programs a part of the curriculum to spread throughout all education years.

### 4.1 Limitations and future directions

The main limitation of the present research could be the fact that the sample contains teachers working in the public primary schools in Amasya city center in the 2018–2019 academic year. Thus, it is suggested that other research be carried out to investigate the health-promoting lifestyle behaviors of teachers at other levels in public and private schools across the country.

## 5 Conclusion

Based on the findings of this research, the results have important implications for teachers working in public primary schools. As a result of this study, it was revealed that the efforts of female teachers working in primary schools to exercise were not sufficient. It has been determined that teachers who are female, single, take courses on health promotion, follow programs and articles about healthy living in written and visual media lead a healthier lifestyle than other groups. It has been revealed that teachers' taking courses on health promotion, and following programs and articles about healthy living in written and visual media affect their healthy lifestyle behaviors. Regarding the result of six components, it was noticed that the highest mean score percentage of health promotion lifestyle behaviors was found for teachers response to the spiritual growth component, followed by the interpersonal relationships component, while teachers' health promotion lifestyle to physical activity component was the lowest. In line with these results, it is recommended to make plans to introduce health promotion lifestyle behaviors, especially exercise, to all teachers, especially female teachers.

## Data availability statement

The original contributions presented in the study are included in the article/supplementary material, further inquiries can be directed to the corresponding author.

## Ethics statement

The study involving human participants was approved by the Amasya Provincial Directorate of National Education. The participants provided written informed consent for participation in the study.

## Author contributions

MÖ: Conceptualization, Data curation, Formal analysis, Funding acquisition, Investigation, Methodology, Project administration, Resources, Software, Supervision, Validation, Visualization, Writing – original draft, Writing – review & editing.
